# The Tetraindole SK228 Reverses the Epithelial-to-Mesenchymal Transition of Breast Cancer Cells by Up-Regulating Members of the miR-200 Family

**DOI:** 10.1371/journal.pone.0101088

**Published:** 2014-06-26

**Authors:** Chie-Hong Wang, Chia-Ling Chen, Shivaji V. More, Pei-Wen Hsiao, Wen-Chun Hung, Wen-Shan Li

**Affiliations:** 1 Institute of Chemistry, Academia Sinica, Taipei, Taiwan; 2 Institute of Biomedical Sciences, National Sun Yat-sen University, Kaohsiung, Taiwan; 3 National Institute of Cancer Research, National Health Research Institutes, Tainan, Taiwan; 4 Agricultural Biotechnology Research Center, Academia Sinica, Taipei, Taiwan; 5 Doctoral Degree Program in Marine Biotechnology, National Sun Yat-Sen University, Kaohsiung, Taiwan; National Cancer Center, Japan

## Abstract

The results of recent studies have shown that metastasis, the most common malignancy and primary cause of mortality promoted by breast cancer in women, is associated with the epithelial-to-mesenchymal transition (EMT). The results of the current study show that SK228, a novel indole containing substance, exhibits anti-cancer activity. In addition, the effects of SK228 on the regulation of EMT in breast cancer cells as well as the underlying mechanism have been explored. SK228 was observed to induce a fibroblastoid to epithelial-like change in the appearance of various breast cancer cell lines and to suppress the migration and invasion of these cancer cells *in* *vitro*. Moreover, expression of E-cadherin was found to increase following SK228 treatment whereas ZEB1 expression was repressed. Expression of other major EMT inducers, including ZEB2, Slug and Twist1, is also repressed by SK228 as a consequence of up-regulation of members of the miR-200 family, especially miR-200c. The results of animal studies demonstrate that SK228 treatment leads to effective suppression of breast cancer growth and metastasis *in* *vivo*. The observations made in this investigation show that SK228 reverses the EMT process in breast cancer cells via an effect on the miR-200c/ZEB1/E-cadherin signalling pathway. In addition, the results of a detailed analysis of the *in* *vivo* anti-cancer activities of SK228, carried out using a breast cancer xenograft animal model, show that this substance is a potential chemotherapeutic agent for the treatment of breast cancer.

## Introduction

The epithelial-to-mesenchymal transition (EMT) of cells is a reversible process that occurs during embryonic development and as a result of pathological events such as fibrosis and cancer progression [1]. For most epithelial tumors, progression toward malignancy is associated with a loss of epithelial differentiation and a shift towards a mesenchymal phenotype. Epithelial cells undergoing EMT lose their cell-cell adhesion ability as well as other epithelial traits, and they acquire migratory and invasive phenotypes that provide cancer cells with greater invasive and metastatic properties.

The results of recent studies show that EMT is an important step in cancer cell metastasis [1], [2]. EMT can be triggered by a number of extracellular factors involving Wnt, transforming growth factor-β (TGF-β), epidermal growth factor (EGF) and insulin-like growth factor (IGF) [3]–[5]. A common hallmark of EMT is the loss of E-cadherin [6], [7], which is highly expressed in epithelial tissues and located at adherens junctions to maintain epithelial integrity. During tissue remodelling or tumor progression, several transcriptional repressors, such as Snail, Slug, ZEB1, ZEB2 and Twist1, can down-regulate E-cadherin expression leading to a reduction of cell-cell adhesion and promotion of EMT and cell motility [8]–[11].

MicroRNAs (miRNAs) are small non-coding RNA molecules that down-regulate gene expression via post-transcriptional mechanisms. It has been observed that miRNAs are involved in the regulation of many physiological and pathological processes through their down-regulation of specific target genes promoted by binding to the 3′UTR of target mRNAs. This process leads to translational repression or mRNA cleavage. miRNAs play important roles in cell proliferation, differentiation, apoptotic cell death, stress resistance, and physiological metabolism [12]. Emerging evidence exists that miRNAs serve as key regulators in the development and progression of cancer [13], [14]. Members of the miR-200 family have been found to act as tumor suppressor miRNAs that inhibit EMT by down regulating the expression of ZEB1 and ZEB2 [15], [16].

The results of recent efforts have demonstrated that several natural products can regulate the expression of miRNA in different types of cancer cells [17], [18]. For example, broccoli-derived compounds like the 3,3′-diindolylmethane (DIM), up-regulate the expression of members of the miR-200 family leading to reversal of the EMT process in pancreatic cancer cells [18]. In an earlier investigation, we showed that tetraindole (1,4-bis{di[1*H*-indol-3-yl]methyl}benzene) and its 5-hydroxy tetraindole derivative, SK228, which contains a benzene core structure with four appended hydroxyindole groups, induce G2 arrest and apoptosis in human breast adenocarcinoma (MCF 7 and MDA-MB-231) cells through externalization of membrane phosphatidylserine, DNA fragmentation, and activation of caspase-3 [19]. In addition, it also has been shown that SK228 treatment causes a marked inhibition of the growth of an A549 tumor cell xenograft without producing adverse effects on liver and kidney function of treated mice [20].

In the study described below, we assessed the effect of SK228 on the invasiveness of human breast cancer cells. In addition, the possibility that SK228 alters the expression profile of miRNAs and induces the reversal of EMT was explored along with the underlying mechanisms for both processes. Finally, a detailed analysis of the *in* *vivo* anti-cancer activity of SK228 was carried out using a breast cancer xenograft animal model.

## Materials and Methods

### Cell culture

MDA-MB-231 human breast cancer cell lines were obtained from the American Type Culture Collection (ATCC) and maintained in L15 medium without CO_2_ at 37°C in a humidified incubator. BT-549 human breast cancer cells were obtained from the American Type Culture Collection (ATCC) and maintained in RPMI-1640 medium supplemented with 0.023 IU/mL bovine insulin and 5% CO_2_ at 37°C in a humidified incubator. Hs-578 T human breast cancer cells were obtained from the Bioresource Collection and Research Center (BCRC) and maintained in DMEM medium supplemented with 0.01 mg/mL bovine insulin and 5% CO_2_ at 37°C in a humidified incubator. All culture media were supplemented with 10% fetal bovine serum, 2 mM L-glutamine, 100 µg/mL streptomycin, 0.25 µg/mL amphotericin B, and 100 U/mL penicillin G.

### Cell viability

The effects of SK228 on cell viability were assessed by using the 3-(4,5-dimethylthiazol-2-yl)-2,5-diphenyl-2H-tetrazolium bromide (MTT, Sigma-Aldrich, St. Louis, MO) assay. Briefly, cancer cells were grown in growth medium with/without SK228 for 24 h. At the end of treatment, cells were harvested for transwell assay and MTT assay. MTT assays were performed by adjusting the serum concentration to mimic the conditions in transwell assays. After 24 h, the medium was removed, replaced by 100 µL of 1 mg/mL of MTT in the culture medium, and cells were incubated in the incubator at 37°C for 4 h. Supernatants were carefully removed without disturbing the pellet and the reduced MTT was solubilized in 100 µL/well DMSO. The absorbance was determined at 570 nm on a plate reader.

### Protein extraction and Western blot analysis

Cells were treated with various concentrations of SK228 for 24 or 48 h. After treatment, cells were collected and lysed in PRO-PREPTM Protein Extraction solution (iNtRON Biotechnology). The lysates were incubated on ice for 20 min and centrifugated at 13,000 g for 30 min. Protein concentrations were measured by using protein assay reagents (Pierce). Equal amounts of total protein were mixed with 4X sample loading buffer (62.5 mM Tris-HCl pH 6.8, 10% glycerol, 20% SDS, 2.5% bromophenol blue, and 5% 2-mercaptoethanol), boiled for 10 min and then fractionated by using electrophoresis on 8% or 10% SDS-PAGE. A pre-stained protein ladder (Fermentas) was used as the molecular weight standard. Proteins were electrically transferred to PVDF membranes and treated for 1 h at room temperature with 0.05% TBST and 5% non-fat milk. Blots were subsequently incubated at 4°C overnight with primary antibodies. Immunoreactive proteins were detected after incubation with horseradish peroxidase-conjugated secondary antibody (PerkinElmer) for 1 h at room temperature. The immunoblots were visualized by using enhanced chemiluminescence (Perkin Elmer). All antibodies used are provided in Table S1.

### RNA isolation and reverse transcription-PCR

RNA was isolated from cultured cells by using TRIzol reagent (Invitrogen) according to the manufacturer’s instructions. Purified RNA was dissolved in nuclease-free water and quantified based on UV absorbances at 260 nm. The purity of total RNA was determined by measuring A260/A280 and A260/A230 ratios. Total RNA samples from cells treated with SK228 were collected for RT-PCR analysis. The expressions of several genes (E-cadherin, N-cadherin, vimentin, Snail1/2, ZEB1/2, and Twist1) were determined. Briefly, 1 µg of total RNA from each sample was subjected to reverse transcription-PCR with Super 2 RT-PCR Mix (PROTECH) according to manufacturer’s protocol in a final volume of 20 µL. The cDNA synthesis reaction conditions were 45°C for 30 min and 95°C for 5 min. The progress of reactions was followed by using the polymerase chain reaction. The PCR reaction conditions were as follows: denaturation at 95°C for 30 sec, annealing at 60°C for 30 sec and extension at 72°C for 1 min. Thirty amplification cycles were performed. The products were separated in 2% agarose gel and detected using UV. GAPDH was also detected as internal control. All cDNA were amplified by PCR using the following primers (Table S2).

### Quantification of miRNA by using real-time reverse transcription-PCR

RNA was extracted from cells using TRIzol reagent (Invitrogen) according to manufacturer’s instructions. For reverse transcription reactions, 1 µg of purified RNA and MMLV High Performance Reverse Transcriptase (EPICENTRE Biotechnologies) were used. The specific miRNAs of interest (miR-200a, miR-200b, and miR-200c) and the internal control, RNU6B, were verified by using Universal Probe Library Technology (Roche) according to manufacturer’s protocol. Real-time PCR reactions were carried out with 20 µL of the reaction mixture in LightCycler 480 Instrument II (Roche). The PCR program was initiated by treatment for 10 min at 95°C before 45 thermal cycles were performed, each for 10 s at 95°C, 30 s at 60°C and 10 s at 72°C. Data were analyzed by using the comparative Ct method. The sequences of real-time primers and probes used in this study are provided in Table S3.

### *In vitro* Migration and invasion assay

Human breast cancer cells were treated with different concentrations of SK228 or 0.1% DMSO control for 24 h, suspended in serum-free medium and transferred onto transwells (BD Biosciences). The lower chamber of each was filled with culture medium supplied with 10% FBS. After 24 h incubation, cells in the top chamber were removed by using a cotton swab. The membrane-trapped cells were fixed, stained with crystal violate and counted employing a light microscope. For the *in* *vitro* invasion assay, human breast cancer cells were suspended in serum-free medium and transferred onto transwells, which were pre-coated with growth factor reduced Matrigel (BD Biosciences). The lower chamber was filled with culture medium supplied with 10% FBS. After 24 h incubation, cells in the top chamber were removed by using a cotton swab. The membrane-trapped cells were fixed and stained with crystal violate, then counted employing a light microscope. Results were collected from three independent experiments.

### Immunofluorescence staining

Human breast cancer cells were seeded on cover slips (BD Biosciences) and treated in the presence or absence of SK288 for 24 h. For E-cadherin staining, cells were fixed in methanol/acetone (1∶1) and probed with FITC-anti-E-cadherin antibody (1∶500; BD Biosciences). To detect nuclei, cells were co-stained with propidium iodine (PI; Sigma) and observed utilizing a fluorescence microscope.

### Transfection

Human breast cancer cells were seeded onto the 6-well plates to 60–80% confluent. siRNA (Invitrogen) or miRNA mimics (Invitrogen) were diluted in Opti-MEM medium. Lipofectamine RNAiMAX reagent was also diluted in Opti-MEM medium. siRNAs were added to diluted Lipofectamine RNAiMAX reagent in a 1∶1 ratio and then added to cells. After 72 h, cells were harvested.

### Xenograft study

Athymic nude mice (BALB/c) of 6 weeks of age were obtained from National Laboratory Animal Center (Taipei, Taiwan) and were raised in a specific pathogen-free environment. 4T1/Luc cells (maintained in DMEM) were kindly provided by Dr. Pei-Wen Hsiao (ABRC Laboratory Animal Core Facility, Agricultural Biotechnology Research Center, Academia Sinica, Taiwan) and injected subcutaneously into the right fifth thoracic mammary fat pad at the density of 5×10^5^ cells/100 µL of normal saline. Tumor volumes were measured every week and calculated using the formula V = 1/2×length×(width)^2^. When tumor growth reached *ca.* 70–100 mm^3^, SK228 or the control, consisting of 30% PEG 400 in normal saline, were injected intraperitoneally on the first 3 days to reach a final concentration of 12.5 mg/kg/week in the first week, then two shots at a dose of 12.5 g/kg/week for another 3 weeks (final dose reach to 50 mg/kg/mouse). Tumor growth and spontaneous metastasis of the tumors were observed using an IVIS50 *in* *vivo* imaging system (Xenogen) with firefly D-luciferin substrate (NanoLight) injection until sacrificed. Forty-one days after implantation and initiation of SK228 treatment, animals were sacrificed by either carbon dioxide or cervical dislocation and dissected to obtain tumor samples. For biochemistry and hematology test, blood samples were collected by intracardiac puncture and stored at 4°Cin tube without or with EDTA anti-coagulant. All experiments were completed according to the Institutional Animal Care and Utilization Committee guidelines.

### Ethics statement

The animal studies were conducted in accordance with a protocol approved by the Institutional Animal Care and Utilization Committee of Academia Sinica, Taipei, Taiwan.

### Statistics

Data from three independent experiments are presented as mean ± S.D. Statistical analyses were conducted by using one-way analysis of variance (ONE-WAY ANOVA) with pair wise comparisons conducted by Newman-Keuls test. The findings were considered significant at a p value<0.05.

## Results

### SK228 inhibits migration and invasion activities of human breast cancer cells *in vitro*

**Figure 1:**
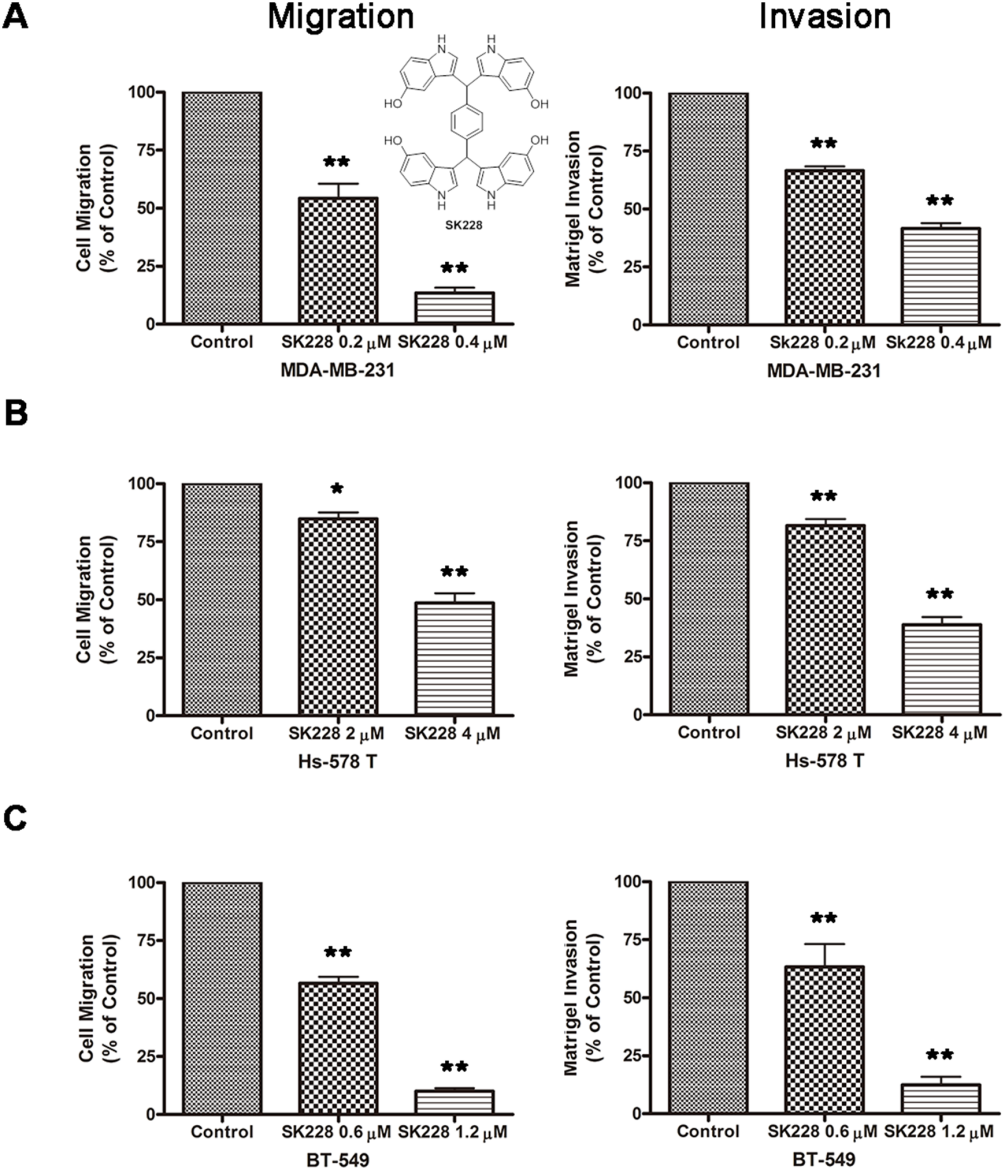
SK228 treatment results in the reduced *in* *vitro* migratory and invasive potential of breast cancer cells. Chemical structure of SK228 is shown. Cells were treated with different concentrations of SK228 or DMSO for 24 h. After incubation, cells were transferred to transwells and given 24 h to migrate or invade. (**A**) Quantitation of the migratory and invasive abilities of MDA-MB-231 cells (**P<0.01). (**B**) Quantitation of the migratory and invasive abilities of Hs-578 T cells (*P<0.05, **P<0.01). (**C**) Quantitation of the migratory and invasive abilities of BT-549 cells (**P<0.01).

The first phase of this investigation focused on the effects of SK228 on the migration and invasion behaviour of breast cancer cells. Three breast cancer cell lines, including MDA-MB-231, Hs-578T and BT-549, all of which exhibit the mesenchymal phenotype, were selected for this study. Treatment with 0.2 µ M SK228 for a 24 h period results in a significant suppression of *in* *vitro* migration and invasion activities of MDA-MB-231 cells (Figures 1A and Figure S1). When 0.2 and 0.4 µM SK228 is present in the transwells used for this purpose, cell migration through the membranes is observed to decrease by 47% and 83%, respectively, compared to that of the untreated control. Cell invasion through Matrigel pre-coated membranes also decreases by 34% and 59% upon respective treatment with 0.2 and 0.4 µ M SK228. SK228 also promotes suppression of the *in* *vitro* migration and invasion activities of Hs-578T and BT-549 cells (Figures 1B and C, Figures S2 and S3). Specifically, when 2 and 4 µM of SK228 are present in the transwells, migration of Hs-578 T cells through the membranes decreases by 16% and 52%, respectively, compared to that of the untreated control. Cell invasion through Matrigel pre-coated membranes also decreases by 19% and 62% upon respective treatment with 2 and 4 µM SK228. Similar results are observed in studies with BT-549 cells.

To exclude the possibility that reduction of migration and invasion abilities of these cancer cells are caused by a growth inhibitory activity of SK228, cell viabilities were concurrently analyzed by using the MTT assay (Figure S4). The results show that no significant differences exist in cell viabilities following 24 h incubation of these cancer cells in the presence or absence of SK228. The data suggest that SK228 suppresses the invasiveness of breast cancer cells in a way that is independent of an effect on growth inhibitory activity.

### SK228 inhibits migration and invasion of breast cancer cells via reversal of EMT

**Figure 2:**
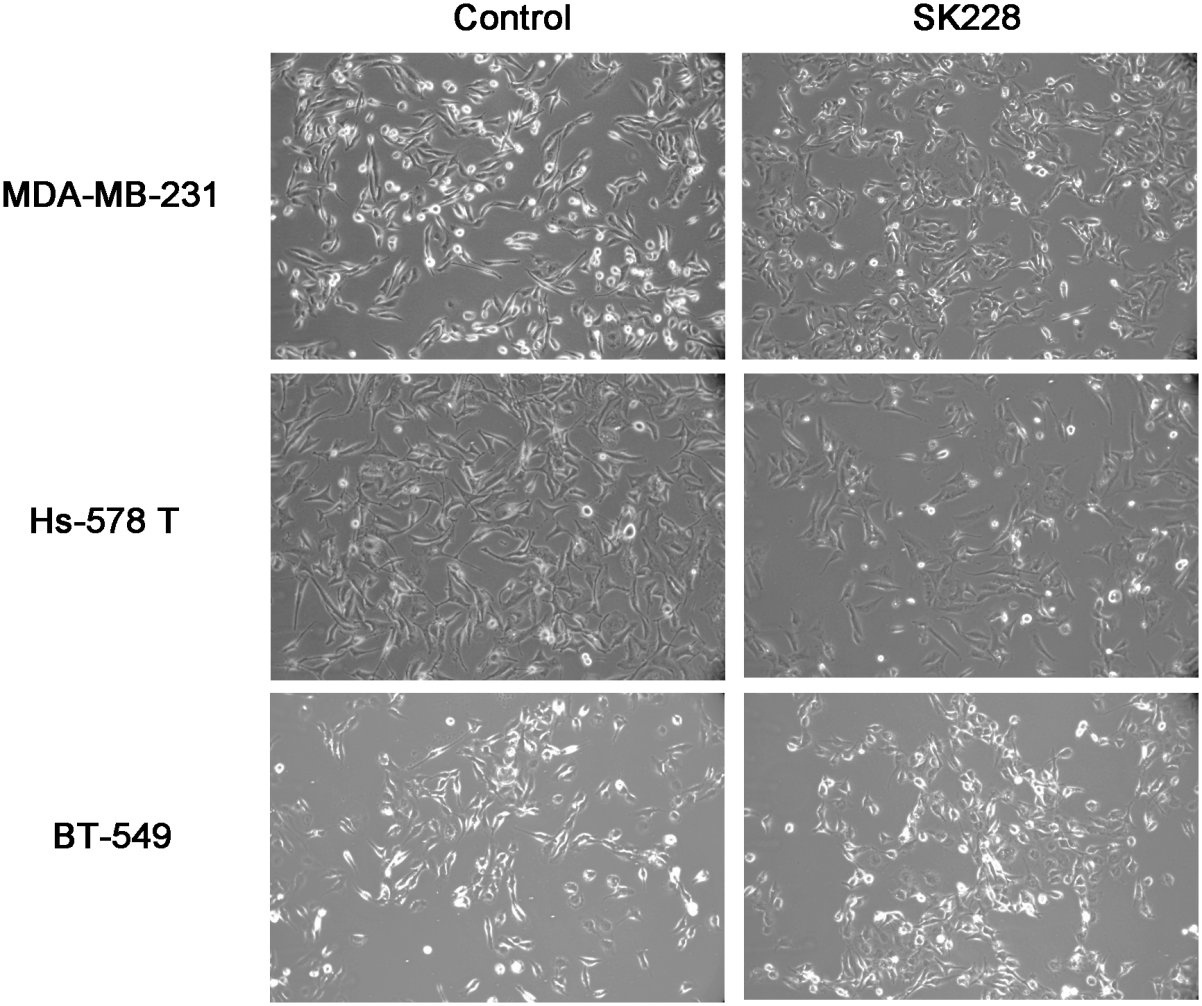
SK228 induces the morphological changes in breast cancer cells. The morphologies of breast cancer cells change from fibroblastoid to epithelial-like after being incubated with SK228 for 24 h (MDA-MB-231 cells: 0.8 µ M of SK228, Hs-578 T cells: 4 µM of SK228 and BT-549 cells: 1.2 µ M of SK228). The effects of SK228 on morphological change were documented by using a light microscopy at the indicated time.

**Figure 3:**
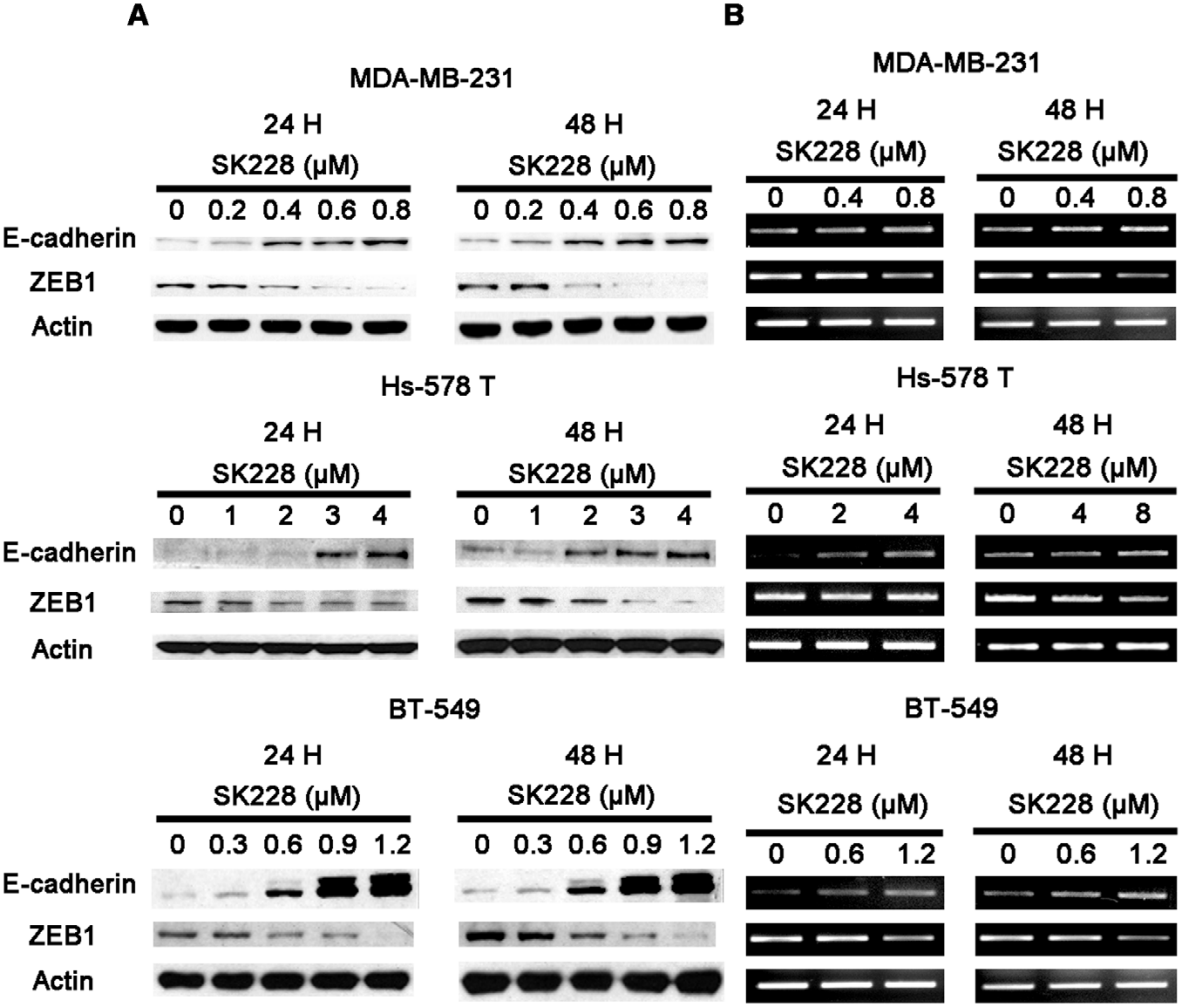
SK228 increases the expression of E-cadherin and decreases the expression of ZEB1 in breast cancer cells. SK228 increases the expression of E-cadherin and decreases the expression of ZEB1 as assessed by using Western Blot analysis (**A**) and RT-PCR (**B**).

Loss or decreased expression of E-cadherin, which has been shown to correlate with invasion and metastasis in a number of malignancies, is also considered to be a crucial step in the progression of EMT. Three human breast cancer cells, which have been reported to express very low levels of E-cadherin and high levels of ZEB1 [21], [22], were employed in a study designed to probe this issue. SK228 is observed to induce the fibroblastoid to epithelial-like morphological changes of these breast cancer cells, suggesting that this substance might regulate expression of E-cadherin (Figure 2) [23] [24]. To evaluate expression of E-cadherin and its transcriptional repressor, ZEB1, in human breast cancer cell lines, protein and mRNA levels were determined by utilizing western blot or reverse-transcriptase PCR methods at 24 and 48 h post SK228 treatment (Figures 3A and B). The results show that the initially low base level of E-cadherin increases in a time- and dose-dependent manner upon treatment with SK228.

**Figure 4:**
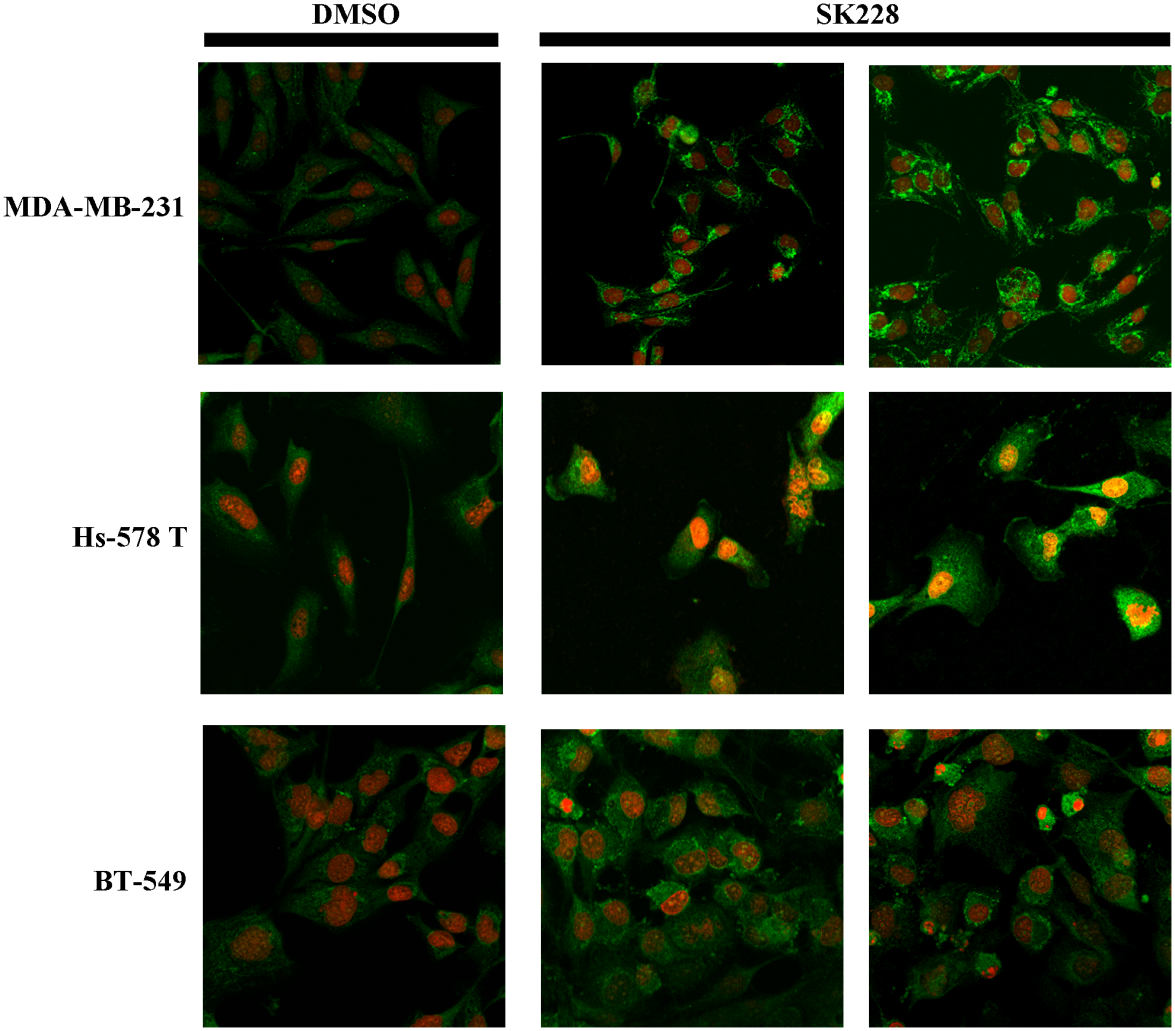
E-cadherin becomes distributed in the cytoplasm closer to the cell membrane after SK228 treatment. Immunofluorescence staining results shows that E-cadherin is induced and distributed in the cytoplasm closer to the cell membrane after SK228 treatment.

In contrast, cancer cell express a high base level of ZEB1, a protein that has been shown to be a crucial promoter of malignant tumor progression [25]. Treatment with SK228 leads to suppression of the expression of ZEB1 at both the protein and mRNA level. Immunofluorescence staining results demonstrate that E-cadherin expression both increases and becomes redistributed in the cytoplasm to locations closer to the cell membrane after SK228 treatment (Figure 4).

The effects of SK228 on the expression of several other EMT inducers or mesenchymal markers, including ZEB2, Snail, Slug, Twist1, N-cadherin and vimentin, were probed (Figures S5, S6 and S7). An earlier report suggested that expression of ZEB2, another ZEB protein family member, inversely correlates with that of E-cadherin [9]. Indeed, we observed that expression of ZEB2 is also repressed by SK228 treatment of breast cancer cells. In contrast, expression of Snail increases after treatment with SK228. Although Snail is known to be required for tumor growth and metastasis, the results of a previous study show that no clear correlation exists between Snail expression and down-regulation of E-cadherin in 20 breast cancer cell lines, including MDA-MB-231 and Hs-578 T cancer cells [26], [27]. We also do not observe an inverse correlation between the expressions of E-cadherin and Snail in the breast cancer cell lines we explored. This finding suggests that Snail does not control the epithelial plasticity of these three breast cancer cells.

Slug, a transcription factor in the Snail family, is also implicated in EMT and the repression of E-cadherin [11], [28]. In contrast to that of Snail, the expression of Slug is suppressed by SK228, a phenomenon that could be caused by Twist1, which has been identified to be a direct inducer of Slug in the promotion EMT [29]. Although the protein expression level of Twist1 in MDA-MB-231 was not explored, the mRNA of Twist1 was observed to decrease upon treatment with SK228. The protein and RNA expression levels of Twist1 were also suppressed in Hs-578 T and BT-549 cells.

Vimentin is a well-documented mesenchymal marker (reviewed in [30]) and its over-expression results in an increase in the motility invasiveness of breast cancer cells [31]. However, the results of the current investigation show that vimentin expression is not affected by SK228. It has been reported that siRNA knockdown of the expression of ZEB1 does not change the level of vimentin protein expression [32]. In addition, we observed that SK228 does not affect the expression of N-cadherin, another mesenchymal marker reported to promote motility, invasion, and metastasis [33].

### SK228 induces expression of members of the miR-200 family in breast cancer cells

**Figure 5:**
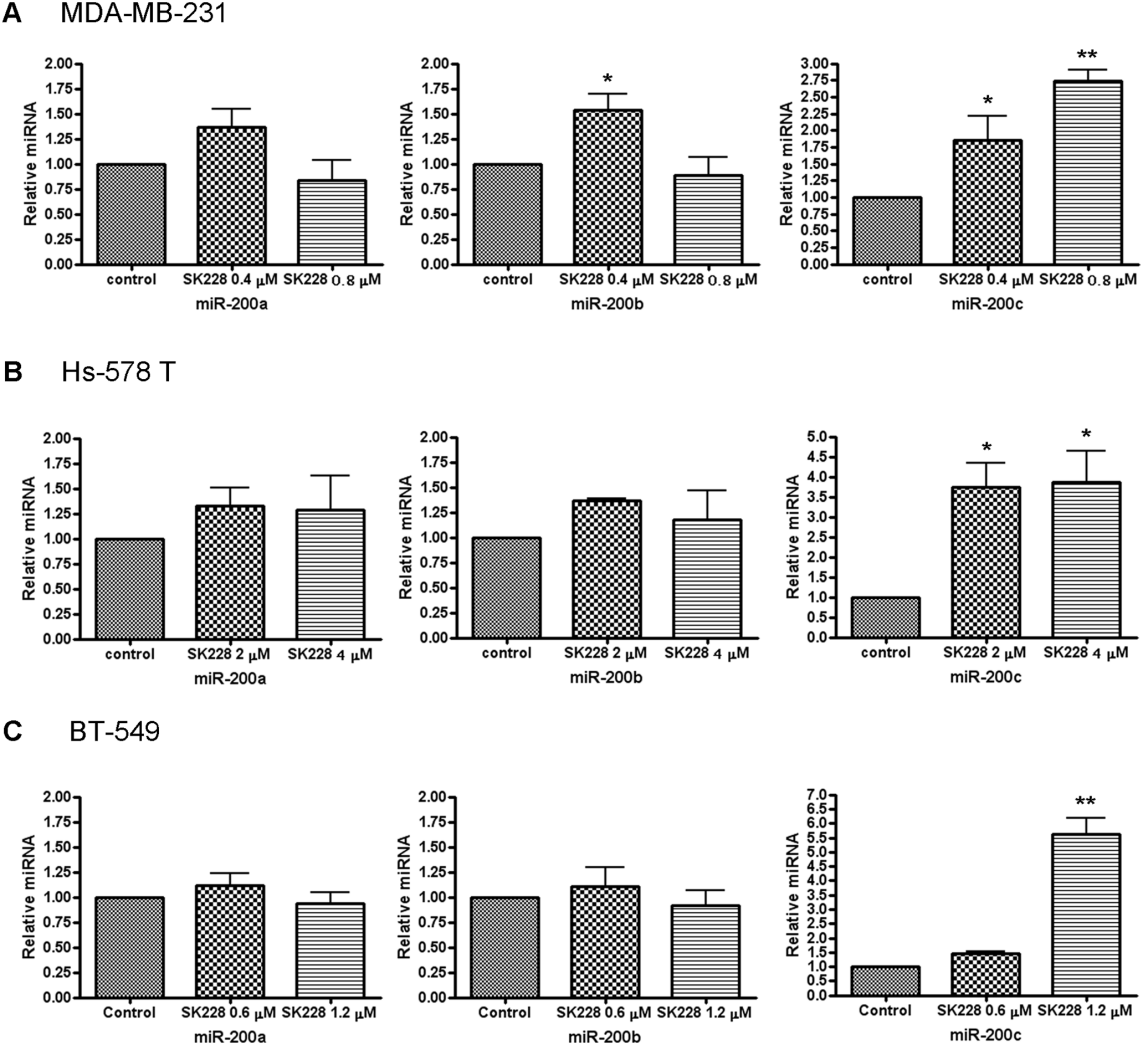
miRNA real-time PCR shows that SK228 induces the exoression of miR-200 family in breast cancer cells. Cells are treated with different concentrations of SK228 for 48-200 family are analyzed by using real-time PCR. (**A**) MDA-MB-231 cells, (**B**) Hs-578 T cells, (**C**) BT-549 cells.

It is well known that members of the miR-200 family cooperatively regulate the E-cadherin transcriptional repressors ZEB1 and ZEB2. Moreover, DIM has been reported to induce the expression of members of the miR-200 family. The possibility that SK228 promotes EMT via a miR-200/ZEB1/E-cadherin pathway was tested by examining the expression profiles of miR-200a, miR-200b and miR-200c in SK228-treated MDA-MB-231, Hs-578 T and BT-549 cells by using real-time PCR. The data arising from this effort demonstrate that SK228 induces expression of members of the miR-200 family, especially miR-200c, in MDA-MB-231 as well as Hs-578 T and BT-549 cells (Figure 5). SK228 promotes a marked dose-dependent increase in the expression of miR-200c, whereas the expression levels of miR-200a and miR-200b are increased by 50% at low doses of SK228 and return to control levels at high doses in MDA-MB-231 and Hs-578 T. Moreover, SK228 appears to specifically modulate expression of miR-200c.

**Figure 6:**
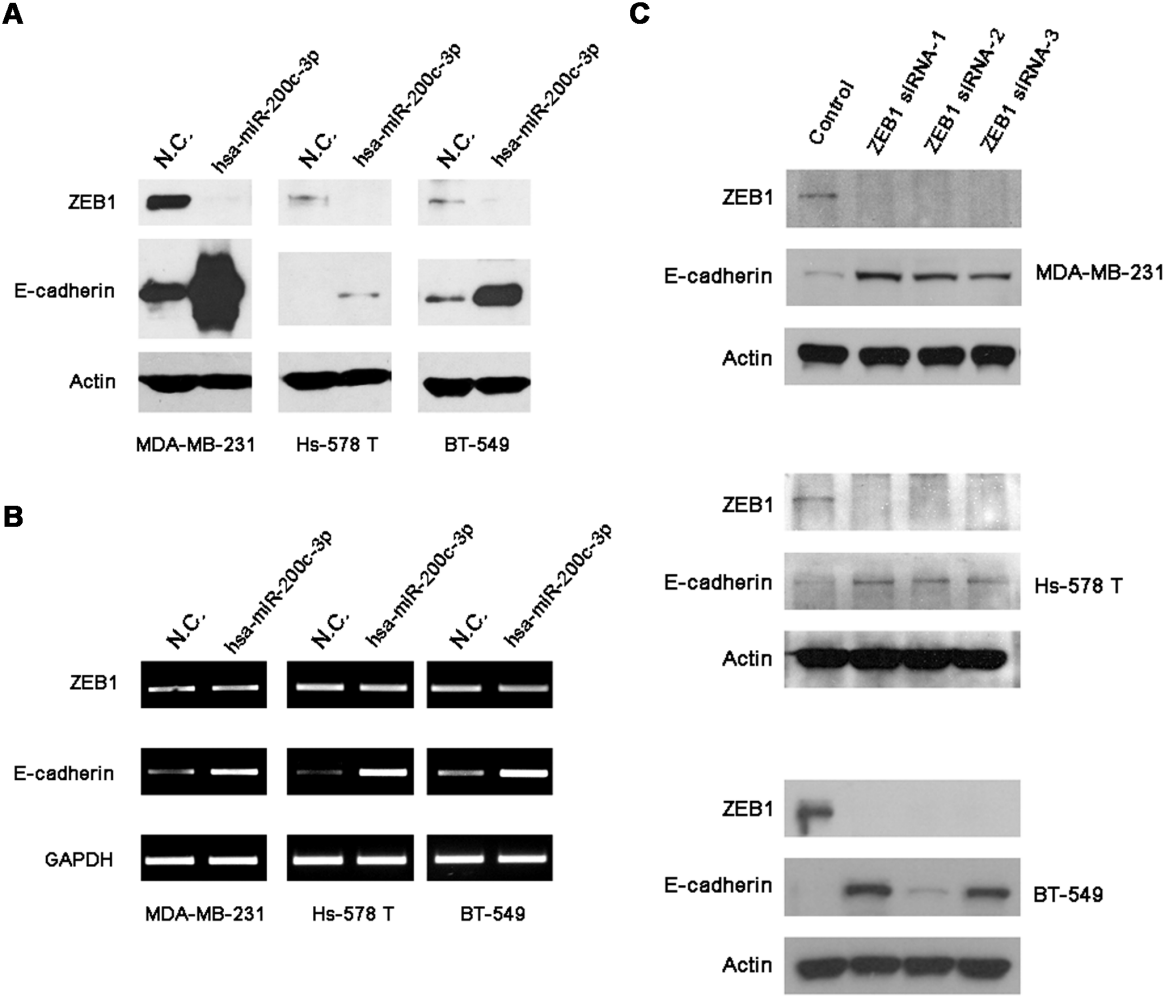
miR-200c/ZEB1/E-cadherin signal pathway regulates the epithelial-to-mesenchymal transition in breast cancer cells. Re-expression of miR-200c in breast cancer cells results in up-regulation of E-cadherin expression and the down-regulation of ZEB1 expressions as assessed by using Western Blot analysis (**A**) and RT-PCR (**B**). siRNA knockdown of the expressions of ZEB1 results in up-regulation of E-cadherin expression in breast cancer cells (**C**).

In order to investigate the role of miR-200c in the reversal of the EMT phenotype of breast cancer cells, pre-miRNA-200c mimics were transfected into the breast cancer cells for 72 h. The results show that re-expression of miR-200c in these cells results in an increase of E-cadherin protein and mRNA levels. ZEB1 protein levels are also markedly suppressed by SK228 but not at mRNA levels, a finding that correlates with miRNA-mediated post-transcriptional inhibition (Figures 6A and B). The morphology of miRNA-200c-transfected breast cancer cells are partially changed from fibroblastoid to epithelial forms (Figure S8). The combined results support our hypothesis that re-expression of miR-200c reverses EMT in the breast cancer cells. To investigate the inhibitory effects of ZEB1 in the three cancer cell lines, RNA interference-mediated gene silencing against ZEB1 was performed and the expression of E-cadherin was determined. As the images displayed in Figure 6C show, gene silencing causes the expressions of ZEB1 to decrease and E-cadherin protein to increase. These observations provide support for the conclusion that ZEB1 is a transcriptional repressor of E-cadherin in these cells and that SK228 regulates the miR-200c/ZEB1/E-cadherin pathway and induces partial reversal of EMT.

### SK228 inhibits the growth of 4T1-Luc xenografts and subsequently delays murine breast cancer cell metastasis

**Figure 7:**
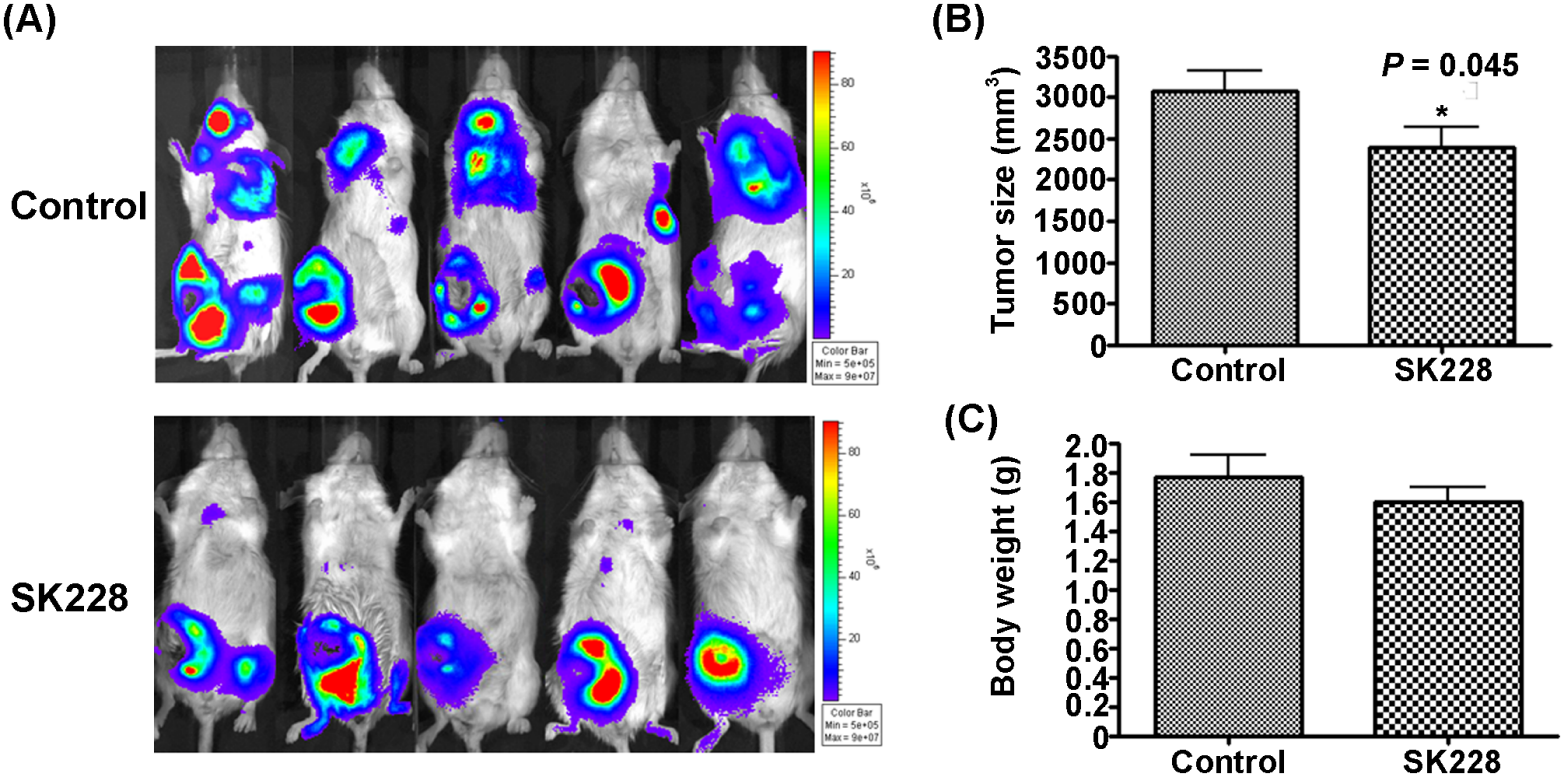
SK228 inhibits the growth of 4T1-Luc xenografts and consequently delays cancer cell metastasis via reversal of EMT process and miR-200c/ZEB1/E-cadherin signaling pathway. (**A**) BALB/c mice bearing the established 4T1-Luc tumors (50 mm^3^) are treated with SK228 (12.5 mg/kg/mouse/week) via intraperitoneal injection on the first 3 days in the first week, then two shots on day 1 and day 4 at a dose of 6.25 mg/kg/week for another 3 weeks (final dose = 50 mg/kg/mouse), or vehicle (30% PEG400+70% PBS) control. The results of spontaneous metastasis assays show that SK228 treatment delays cancer cell metastasis in animal models. (B) The tumor size indicated in the graph represents the measurement of the primary tumor only. Tumor volumes, measured every other day until day 28, are expressed as mean ± SD; *P = 0.045. (C) The average body weight between vehicle and SK228 groups is not significantly different.

The *in* *vivo* therapeutic effect of SK228 on tumor growth and metastasis was studied employing a BALB/c mice model of murine 4T1-Luc mammary adenoma cells. First, 4T1-Luc cells (5×10^5^ cells) were orthotopically injected into the mammary fat pad of BALB/c mice. Then the mice were administered SK228 (12.5 mg/kg/mouse/week) via intraperitoneal injection on the first 3 days in the first week, then two shots on day 1 and day 4 at a dose of 6.25 mg/kg/week for another 3 week period (final dose = 50 mg/kg/mouse). This same procedure was performed using 30% PEG400 in normal saline as control. As the results provided in Figures 7A–B show, the growth of 4T1 cells as reflected by tumour volume is reduced in SK228-treated mice compared with that of control mice (*P* = 0.045). Moreover, no significant loss in body weight occurs in the treated animals (Figure 7C). A particularly significant feature of these observations is that they provide unambiguous evidence that treatment of mice with SK228 results in a profound delay in the metastasis potential of cancer cells (Figure 7A). Thus, the findings suggest that SK228 may be a potent anticancer agent for treatment of human breast cancer.

## Discussion

In the investigation described above, we explored the mechanisms of and targets for EMT modulation by the novel indole derivative SK228. In this effort, we found that SK228 promotes time- and dose-dependent up-regulation of breast cancer cell expression of miR-200c, a member of the miRNA-200 family that have been implicated in EMT and metastasis processes [34], [35]. Several mechanisms could possibly be involved in the miRNA regulation process, including changes in miRNA copy number, polymorphisms and mutations in miRNAs, and transcriptional and epigenetic regulation [36]. Recently, it was observed that the indole derivative, 3,3′-diindolylmethane (DIM), inhibits HDAC activity in prostate cancer cells [37]. We suggest that epigenetic regulation, such as changes in DNA methylation of miRNA gene promoters or chromatin histone deacetylases (HDAC), could be involved in SK228-mediated miRNA regulation.

The results of the current studies also show that SK228 suppresses HDAC activity and reduces the DNA methylation status of miR-200c promoter in MDA-MB-231 cells, which is followed by an increase in the expression of miR-200c (Figures S9 and S10). However, the detailed mechanisms for these outcomes need to be addressed in further studies. miR-200c has also been reported to participate in the modulation of chemoresistance and stem cell-like properties [21], [38]–[40]. Thus, it is possible that SK228 regulates the chemoresistance and stem cell-like properties of breast cancer cells via up-regulating miR-200c.

## Conclusion

Invasiveness is the malignancy hallmark of cancer cells. In this study, we found that the novel tetraindole, SK228, exhibits anti-invasive and anti-migratory activities in human breast cancer cells. SK228 also induces the expressions of E-cadherin in these cells and, moreover, it modulates the expression of miRNA-200 family members (especially miR-200c) and regulates the epithelial-to-mesenchymal transition by targeting ZEB1. Finally and importantly, administration of SK228 causes inhibition of *in* *vivo* xenograft growth and, consequently, it delays cancer cell metastasis in an animal model. Taken together, these results demonstrate that SK228 has a high potential to be a chemotherapeutic agent for breast cancer treatment.

## Supporting Information

Figure S1 SK228 treatment results in reduced *in* *vitro* migratory and invasive potential of MDA-MB-231 cells in a transwell assay. MDA-MB-231 cells treated with SK228 for 24 h show markedly reduced migratory and invasive activities compared with DMSO control. The results were documented with a light microscopy.
(TIF)

Figure S2 SK228 treatment results in reduced *in* *vitro* migratory and invasive potential of Hs-578 T cells in a transwell assay. Hs-578 T cells treated with SK228 for 24 h show markedly reduced migratory and invasive activities compared with DMSO control. The results were documented with a light microscopy.
(TIF)

Figure S3 SK228 treatment results in reduced *in* *vitro* migratory and invasive potential of BT-549 cells in a transwell assay. BT-549 cells treated with SK228 for 24 h show markedly reduced migratory and invasive activities compared with DMSO control. The results were documented with a light microscopy.
(TIF)

Figure S4 The anti-proliferation activities of SK228 in breast cancer cells. No significant differences are found to exist in cell viabilities in the absence and presence of SK228 (>90% viability) during transwell assays, which suggests that the inhibitory effects of SK228 on the cell migration and invasion cannot contribute its cytotoxic effects.
(TIF)

Figure S5 SK228 modulates the expressions of several EMT inducers in MDA-MB-231 cells. ZEB2 and slug were suppressed in both protein and mRNA levels after SK228 treatment. The expression of twist1 protein was not probed but the mRNA was suppressed by SK228. Interestingly while little to no expression of snail occurs in MDA-MB-231 cells, its expression is induced by SK228 treatment. Two mesenchymal markers, vimentin and N-cadherin show no significant changes after SK228 treatment for 48 h.
(TIF)

Figure S6 SK228 modulates the expressions of several EMT inducers in Hs-578 T cells. ZEB2 and slug are suppressed in both protein and mRNA levels by SK228 treatment. The expression of twist1 protein was not probed but the mRNA was suppressed by SK228. Interestingly, while little or no expression of snail occurs in Hs-578 T cells, its expression is induced by SK228. Two mesenchymal markers, vimentin and N-cadherin show no significant changes after SK228 treatment for 48 h.
(TIF)

Figure S7 SK228 modulates the expressions of several EMT inducers in BT-549 cells. ZEB2 and slug were suppressed in both protein and mRNA levels after SK228 treatment. The expression of twist1 protein was not probed but the mRNA was suppressed by SK228. Interestingly, while little or no expression of snail in BT-549 cells, its expression is induced by SK228. Two mesenchymal markers, vimentin and N-cadherin show no significant changes after SK228 treatment at 48 h.
(TIF)

Figure S8 Re-expression of miR-200c leads to a morphological change in breast cancer cells. After transfection with hsa-miR-200c, the morphologies of breast cancer cells changes from fibroblastoid to epithelial-like. This observation is in accordance with SK228 treatment. The effects of miR-200c on morphological change were documented by using a light microscopy at the indicated time.
(TIF)

Figure S9 Effects of SK228 on HDAC activity. After incubation with SK228 for 48 h, nuclear extracts of MDA-MB-231 cells were collected by using a Nuclear Extract kit (Active Motif) and normalized. Histone deacetylase activities were measured by using HDAC Assay kit (Active Motif). The fluorescence of sample was determined by using a plate reader with an excitation wavelength of 360 nm and emission wavelength of 460 nm.
(TIF)

Figure S10 Representative programs of the hsa-miR-200c promoter sequence examined in our study. For methylation analysis of the miR-200c-promoter-specific sequence, purified genomic DNA samples were sent to a service provider (Genomics BioSci & Tech, New Taipei City, Taiwan). The primer was designed by QIAGEN PyroMark Assay Design 2.0 software and DNA conversions were conducted by using QIAGEN EpiTect Plus DNA Bisulfite Kit. For pyrosequencing, the converted samples were analyzed on QIAGEN PyroMark Q24. (A) Control, (B) cells treated with 0.8 µM of SK228 for 48 h, (C) cells treated with 10 µM of AZA (5-Aza-2′-deoxycytidine) for 6 d. The percentages in boxes indicate the individual CpGs methylation values.
(TIF)

Table S1 Information about antibodies used in this study.
(DOCX)

Table S2 Sequences of primers used in this study.
(DOCX)

Table S3 Sequences of real-time primers and probes used in this study.
(DOCX)

Checklist S1 ARRIVE checklist.
(DOCX)
