# Supplementary figures and images for: The Tetraindole SK228 Reverses the Epithelial-to-Mesenchymal Transition of Breast Cancer Cells by Up-Regulating Members of the miR-200 Family
Source: PLoS One. 2014 Jun 26;9(6):e101088. doi: 10.1371/journal.pone.0101088 (PMC4072721; doi:10.1371/journal.pone.0101088)

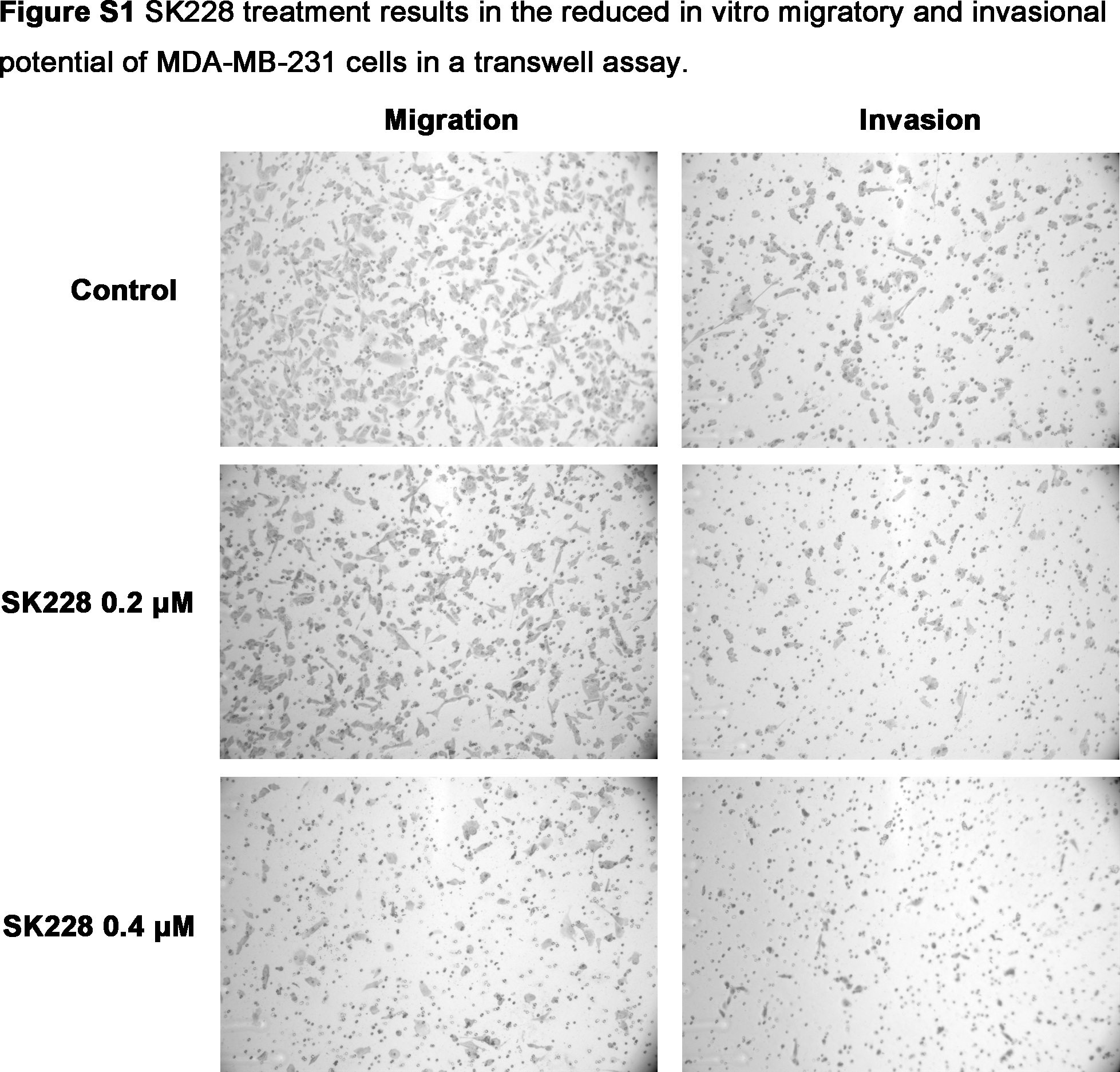

Supplement: Figure S1 — SK228 treatment results in reduced in vitro migratory and invasive potential of MDA-MB-231 cells in a transwell assay. MDA-MB-231 cells treated with SK228 for 24 h show markedly reduced migratory and invasive activities compared with DMSO control. The results were documented with a light microscopy. (TIF) [file pone.0101088.s001.tif]

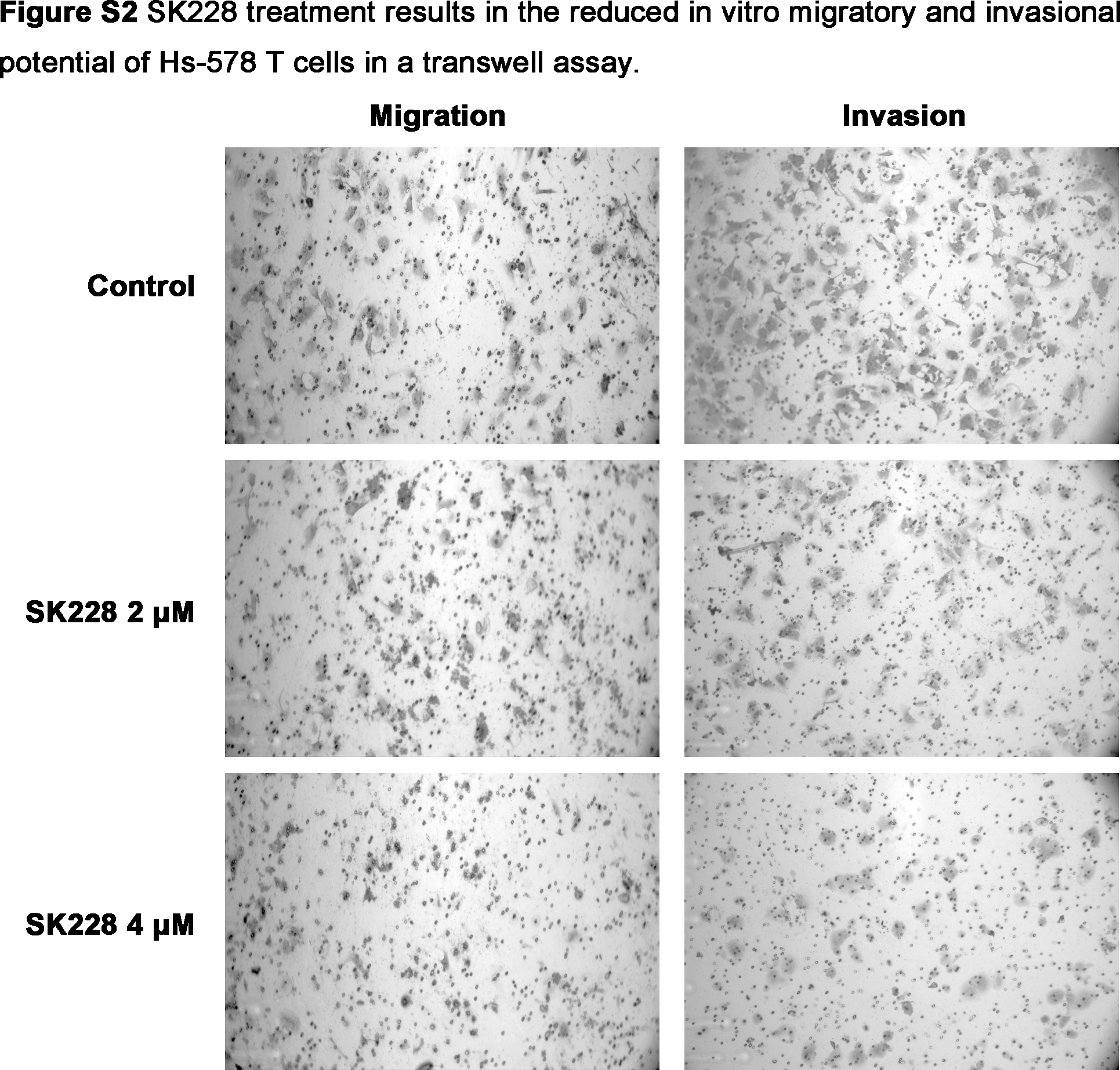

Supplement: Figure S2 — SK228 treatment results in reduced in vitro migratory and invasive potential of Hs-578 T cells in a transwell assay. Hs-578 T cells treated with SK228 for 24 h show markedly reduced migratory and invasive activities compared with DMSO control. The results were documented with a light microscopy. (TIF) [file pone.0101088.s002.tif]

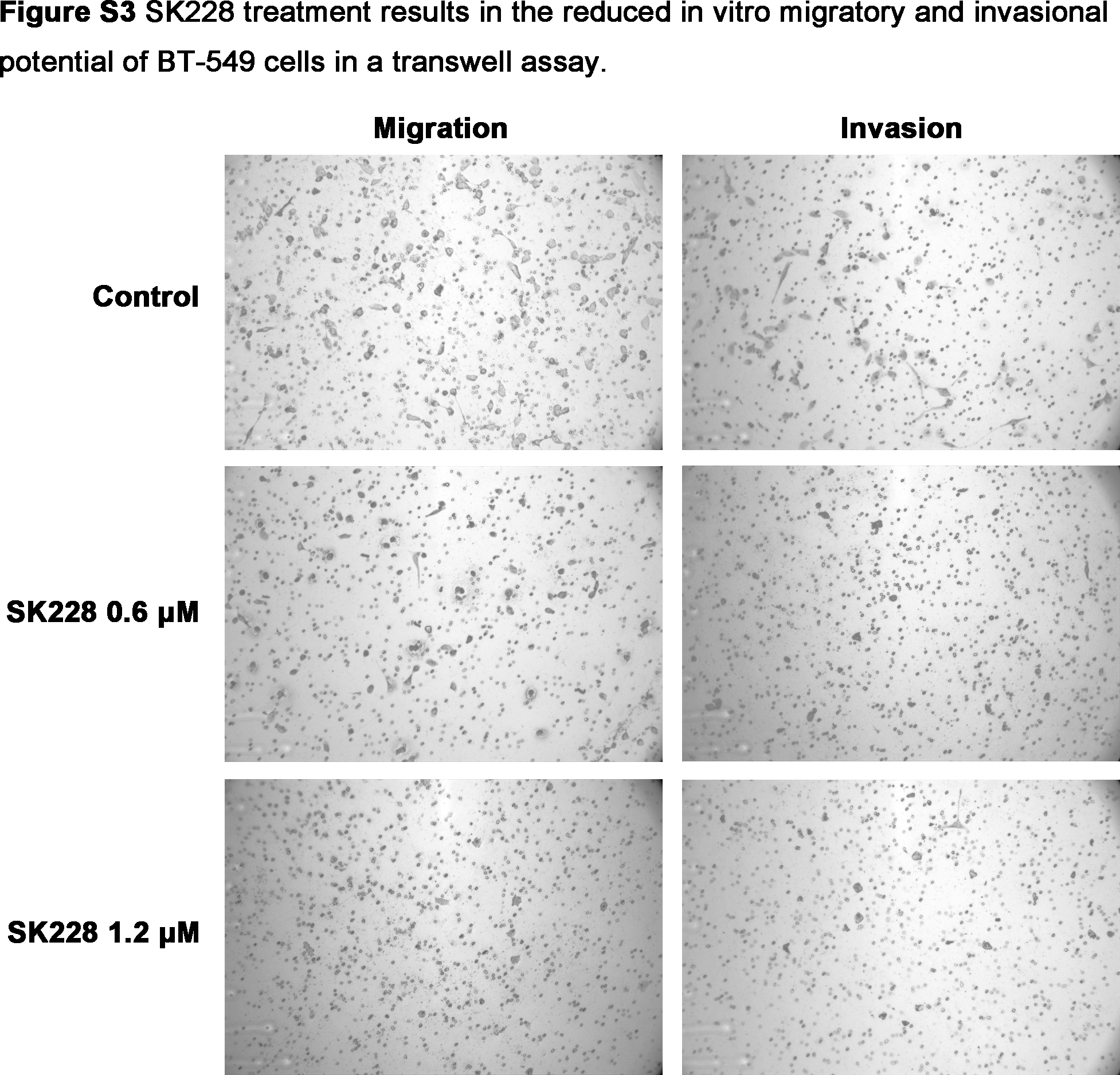

Supplement: Figure S3 — SK228 treatment results in reduced in vitro migratory and invasive potential of BT-549 cells in a transwell assay. BT-549 cells treated with SK228 for 24 h show markedly reduced migratory and invasive activities compared with DMSO control. The results were documented with a light microscopy. (TIF) [file pone.0101088.s003.tif]

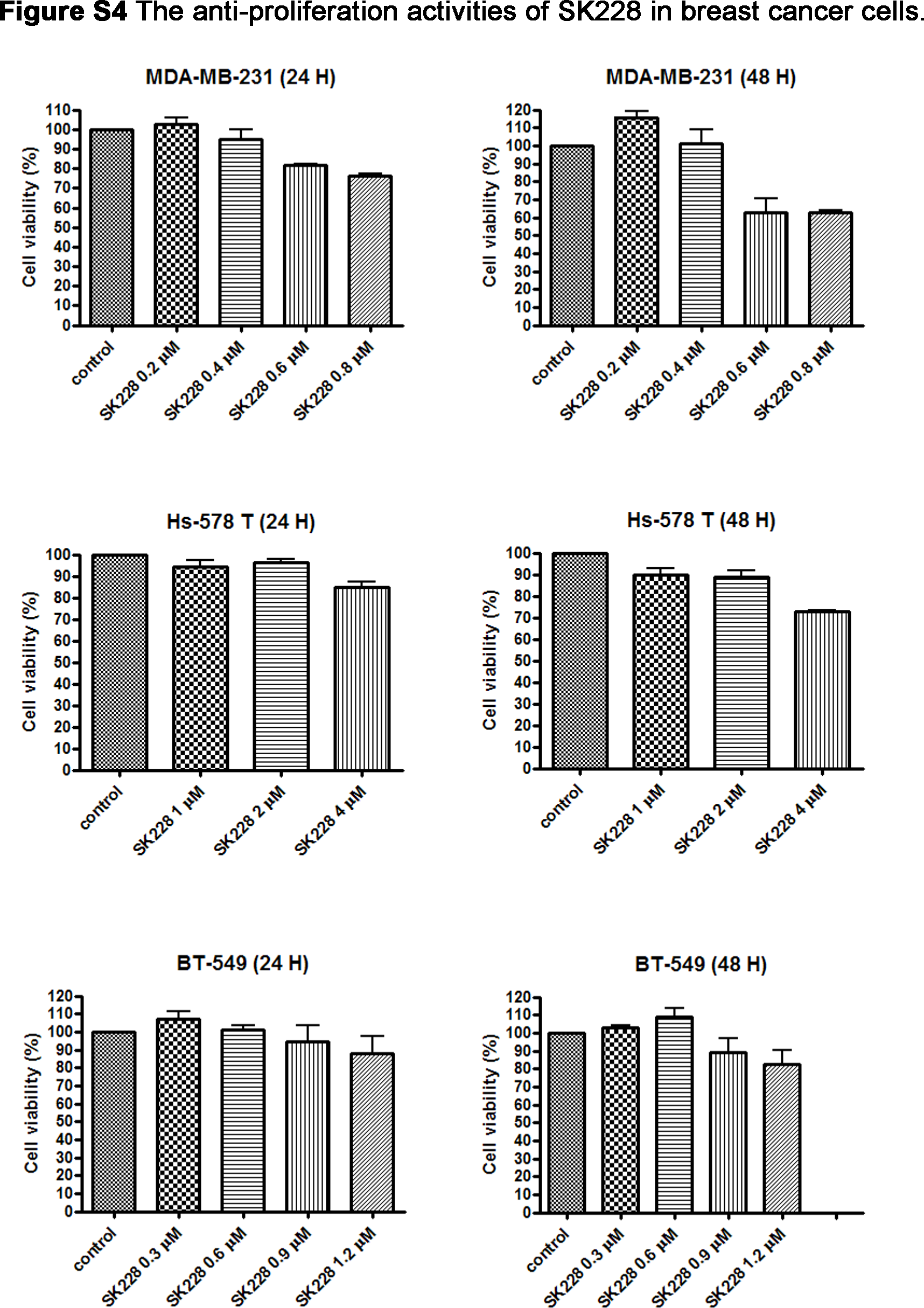

Supplement: Figure S4 — The anti-proliferation activities of SK228 in breast cancer cells. No significant differences are found to exist in cell viabilities in the absence and presence of SK228 (>90% viability) during transwell assays, which suggests that the inhibitory effects of SK228 on the cell migration and invasion cannot contribute its cytotoxic effects. (TIF) [file pone.0101088.s004.tif]

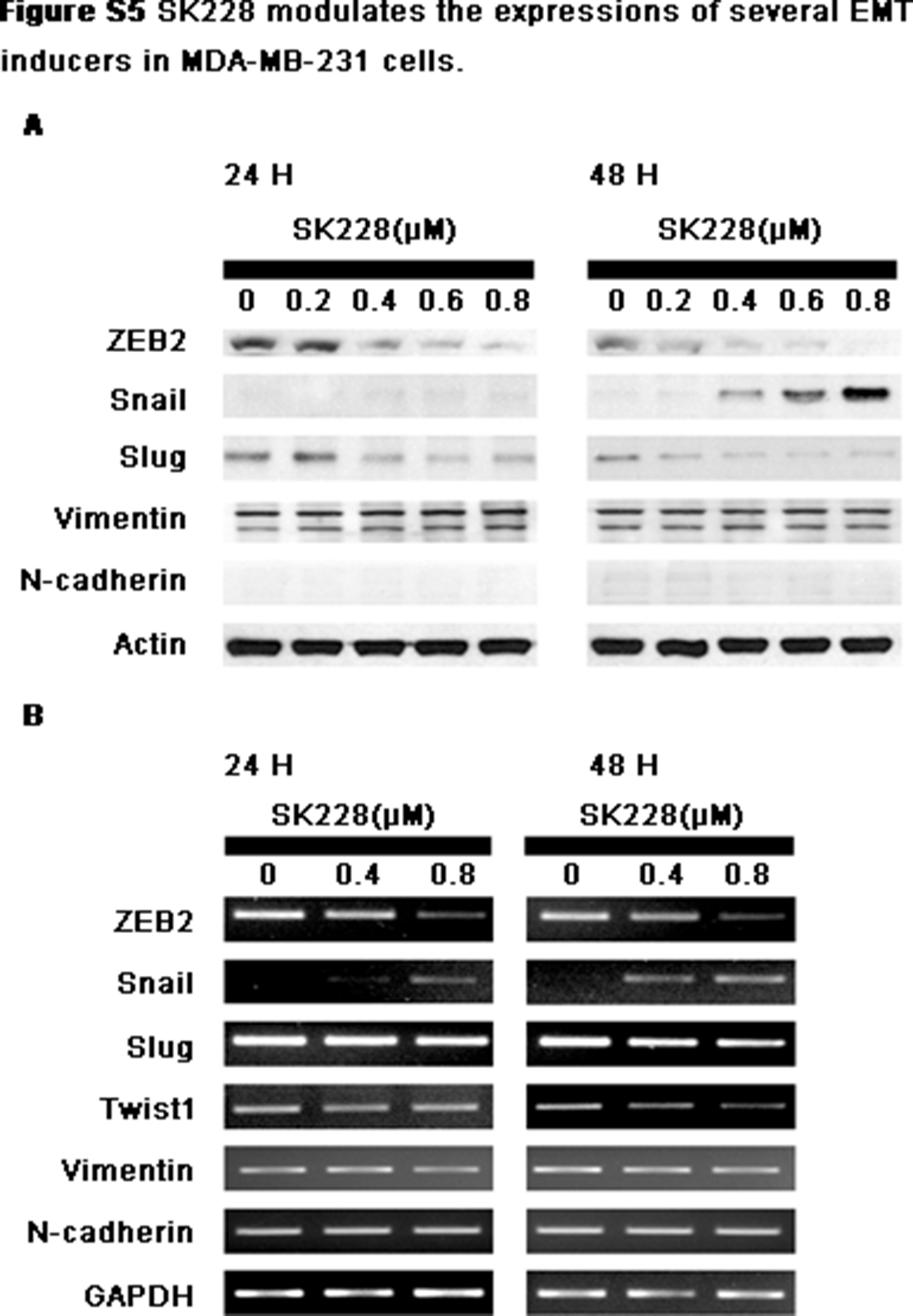

Supplement: Figure S5 — SK228 modulates the expressions of several EMT inducers in MDA-MB-231 cells. ZEB2 and slug were suppressed in both protein and mRNA levels after SK228 treatment. The expression of twist1 protein was not probed but the mRNA was suppressed by SK228. Interestingly while little to no expression of snail occurs in MDA-MB-231 cells, its expression is induced by SK228 treatment. Two mesenchymal markers, vimentin and N-cadherin show no significant changes after SK228 treatment for 48 h. (TIF) [file pone.0101088.s005.tif]

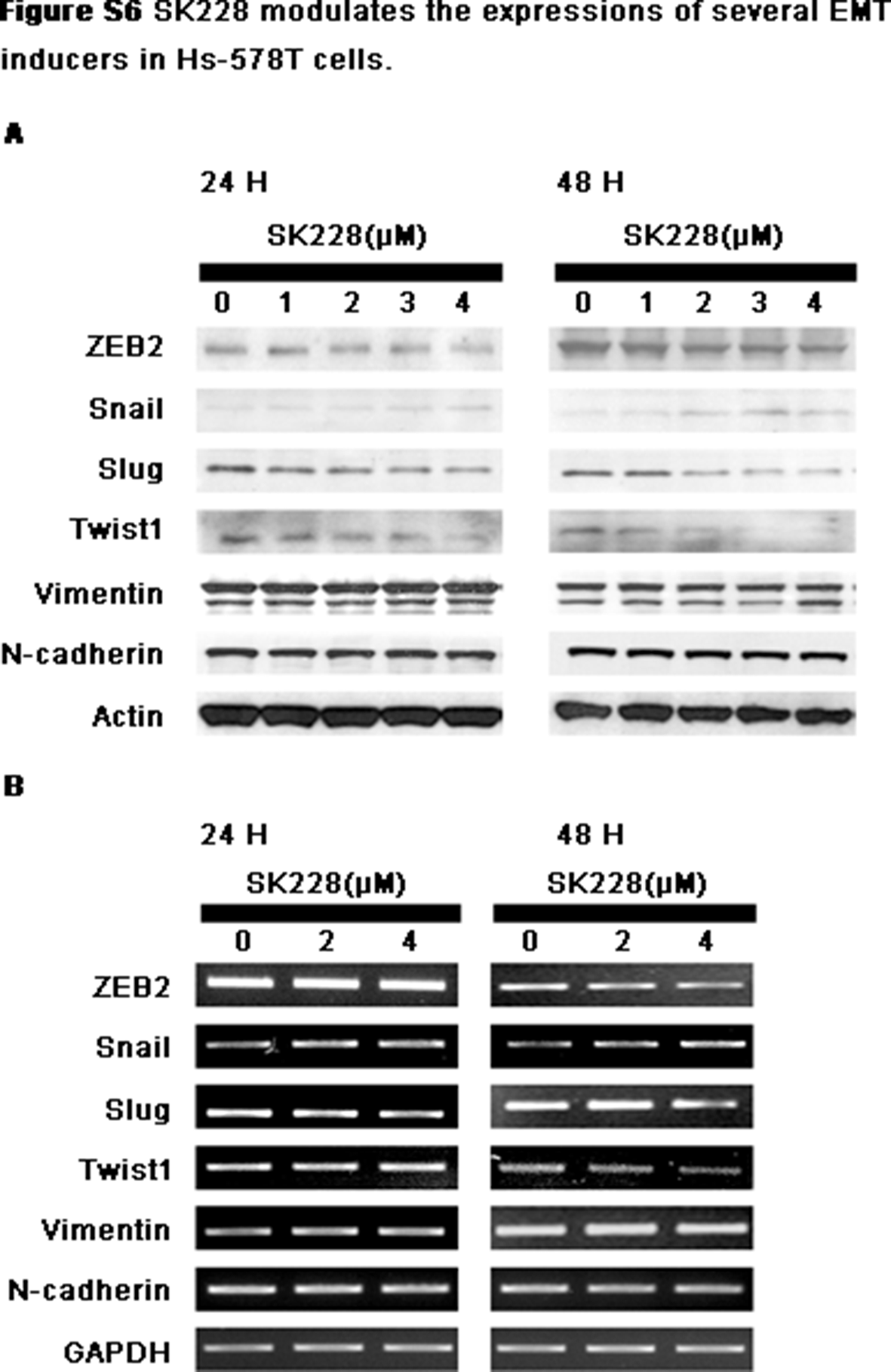

Supplement: Figure S6 — SK228 modulates the expressions of several EMT inducers in Hs-578 T cells. ZEB2 and slug are suppressed in both protein and mRNA levels by SK228 treatment. The expression of twist1 protein was not probed but the mRNA was suppressed by SK228. Interestingly, while little or no expression of snail occurs in Hs-578 T cells, its expression is induced by SK228. Two mesenchymal markers, vimentin and N-cadherin show no significant changes after SK228 treatment for 48 h. (TIF) [file pone.0101088.s006.tif]

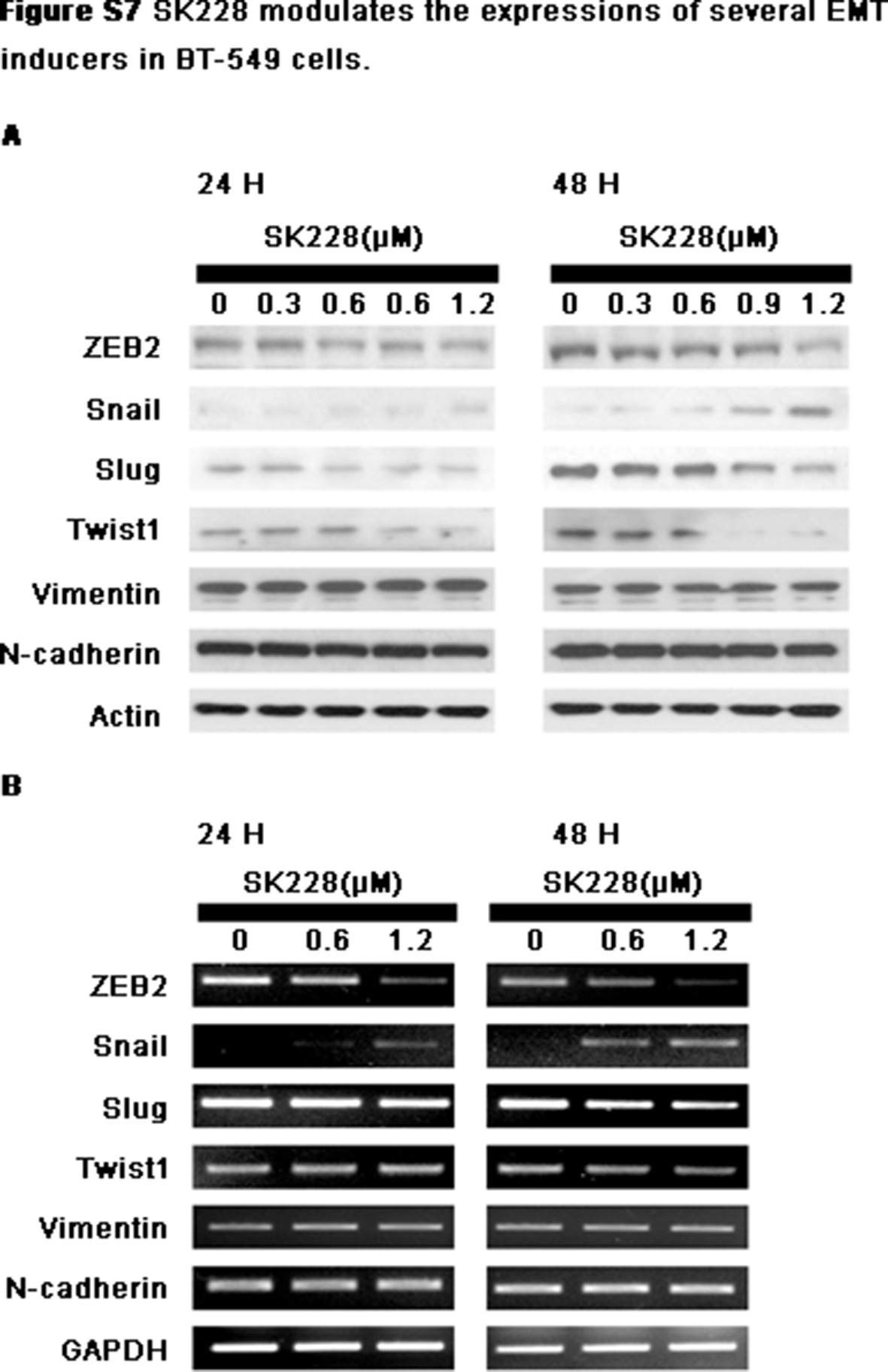

Supplement: Figure S7 — SK228 modulates the expressions of several EMT inducers in BT-549 cells. ZEB2 and slug were suppressed in both protein and mRNA levels after SK228 treatment. The expression of twist1 protein was not probed but the mRNA was suppressed by SK228. Interestingly, while little or no expression of snail in BT-549 cells, its expression is induced by SK228. Two mesenchymal markers, vimentin and N-cadherin show no significant changes after SK228 treatment at 48 h. (TIF) [file pone.0101088.s007.tif]

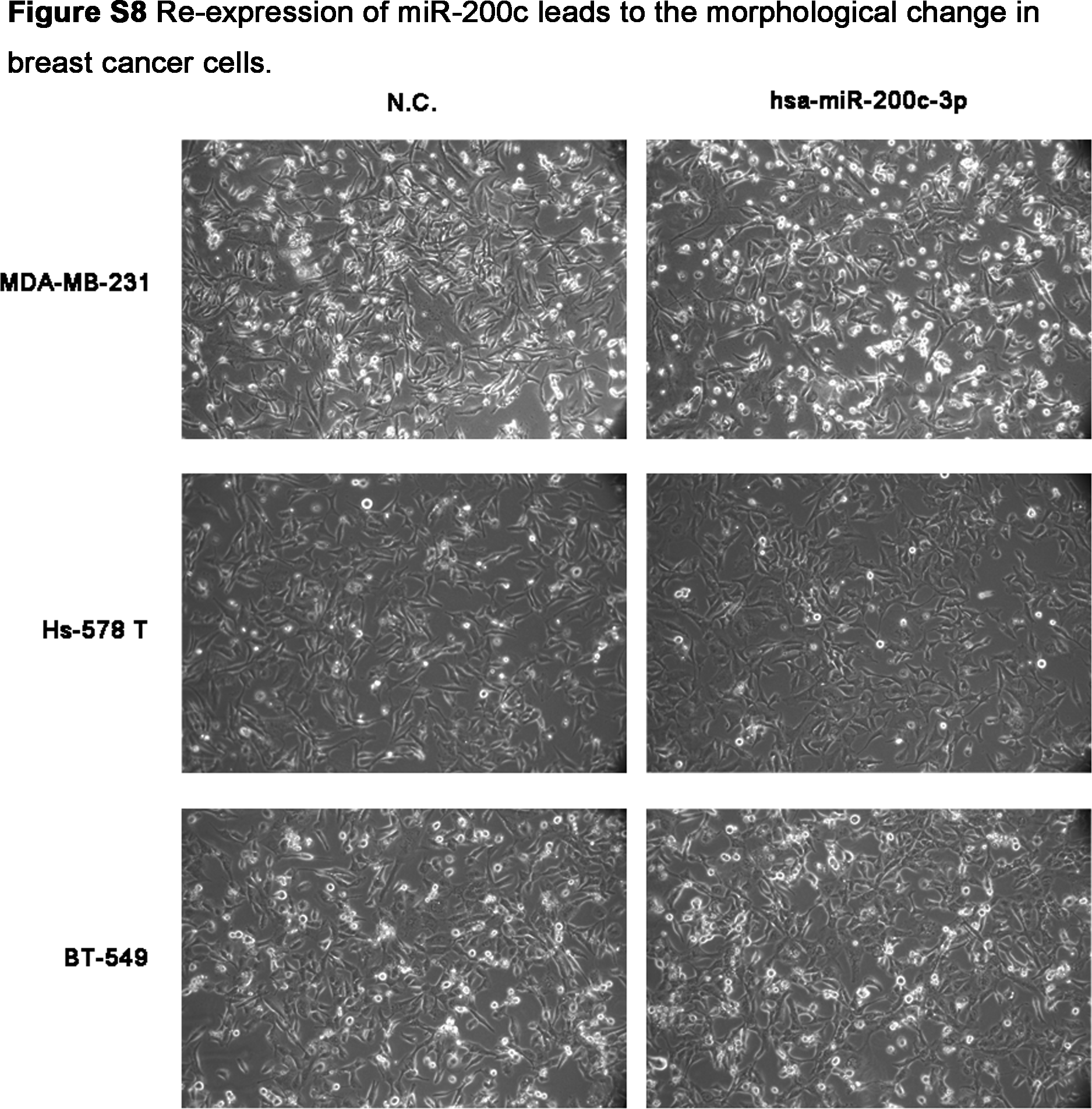

Supplement: Figure S8 — Re-expression of miR-200c leads to a morphological change in breast cancer cells. After transfection with hsa-miR-200c, the morphologies of breast cancer cells changes from fibroblastoid to epithelial-like. This observation is in accordance with SK228 treatment. The effects of miR-200c on morphological change were documented by using a light microscopy at the indicated time. (TIF) [file pone.0101088.s008.tif]

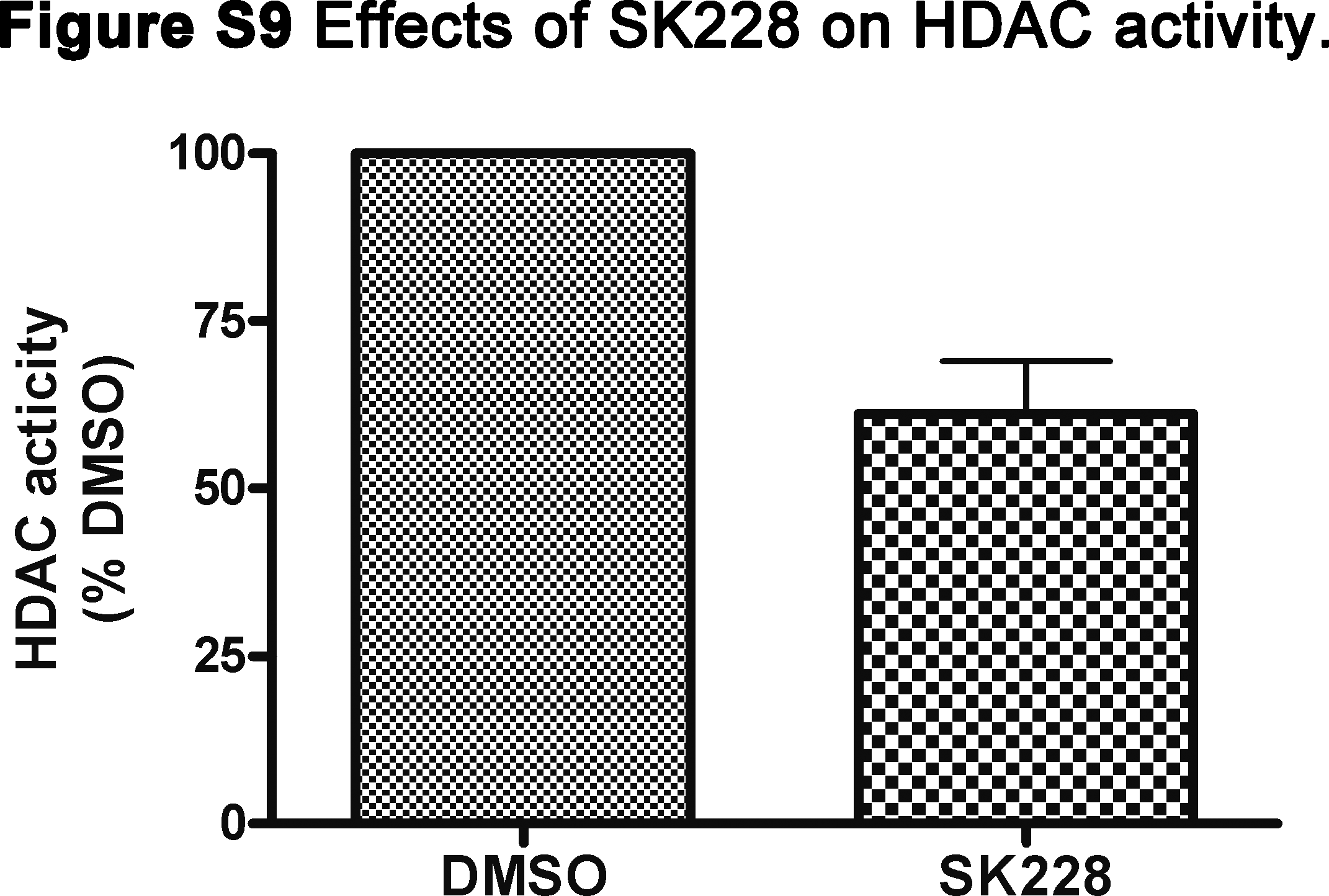

Supplement: Figure S9 — Effects of SK228 on HDAC activity. After incubation with SK228 for 48 h, nuclear extracts of MDA-MB-231 cells were collected by using a Nuclear Extract kit (Active Motif) and normalized. Histone deacetylase activities were measured by using HDAC Assay kit (Active Motif). The fluorescence of sample was determined by using a plate reader with an excitation wavelength of 360 nm and emission wavelength of 460 nm. (TIF) [file pone.0101088.s009.tif]

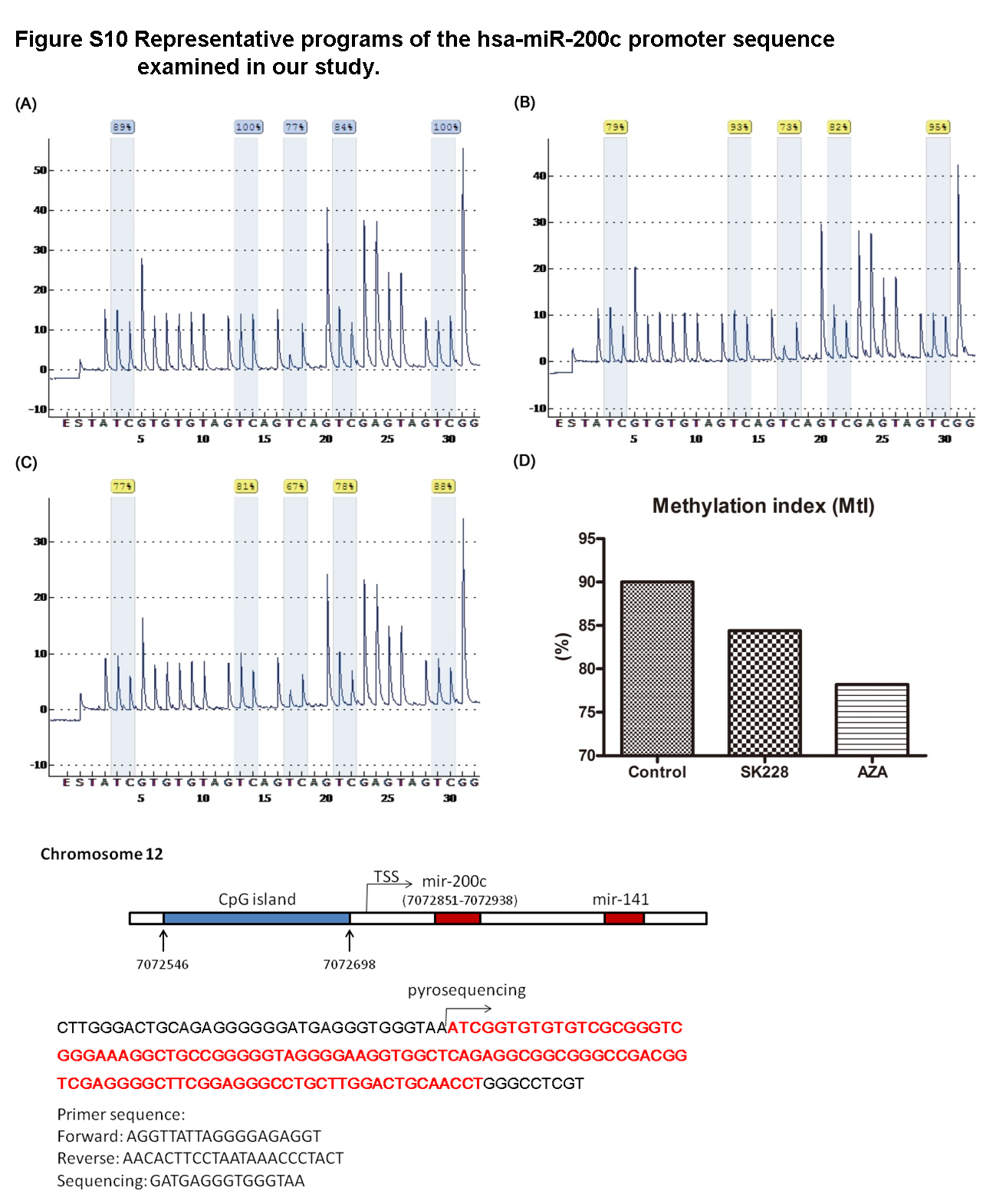

Supplement: Figure S10 — Representative programs of the hsa-miR-200c promoter sequence examined in our study. For methylation analysis of the miR-200c-promoter-specific sequence, purified genomic DNA samples were sent to a service provider (Genomics BioSci & Tech, New Taipei City, Taiwan). The primer was designed by QIAGEN PyroMark Assay Design 2.0 software and DNA conversions were conducted by using QIAGEN EpiTect Plus DNA Bisulfite Kit. For pyrosequencing, the converted samples were analyzed on QIAGEN PyroMark Q24. (A) Control, (B) cells treated with 0.8 µM of SK228 for 48 h, (C) cells treated with 10 µM of AZA (5-Aza-2′-deoxycytidine) for 6 d. The percentages in boxes indicate the individual CpGs methylation values. (TIF) [file pone.0101088.s010.tif]
