# Supplementary material for: The Tetraindole SK228 Reverses the Epithelial-to-Mesenchymal Transition of Breast Cancer Cells by Up-Regulating Members of the miR-200 Family
Source: PLoS One. 2014 Jun 26;9(6):e101088. doi: 10.1371/journal.pone.0101088 (PMC4072721; doi:10.1371/journal.pone.0101088)
Supplement: Table S1 — Information about antibodies used in this study. (DOCX) [file pone.0101088.s011.docx]

## Table S1 The information of antibodies used in this study.

| Antibody | Company | Catalog number |
| --- | --- | --- |
| Actin | Chemicon | MAB1501 |
| Anti-mouse-IgG HRP-labeled | Perkin Elmer | NEF822 |
| Anti-rabbit-IgG HRP-labeled | Perkin Elmer | NEF812 |
| E-cadherin | Cell Signaling | 3195 |
| FITC-E-cadherin | BD | 612130 |
| N-cadherin | BD | 610921 |
| Snail | Cell Signaling | 3879 |
| Slug | Cell Signaling | 9585 |
| Twist | Active Motif | 61097 |
| Vimentin | Cell Signaling | 3932 |
| ZEB1 | Cell Signaling | 3396 |
| ZEB2 | GeneTex | GTX85180 |
