# Supplementary material for: The Tetraindole SK228 Reverses the Epithelial-to-Mesenchymal Transition of Breast Cancer Cells by Up-Regulating Members of the miR-200 Family
Source: PLoS One. 2014 Jun 26;9(6):e101088. doi: 10.1371/journal.pone.0101088 (PMC4072721; doi:10.1371/journal.pone.0101088)
Supplement: Table S2 — Sequences of primers used in this study. (DOCX) [file pone.0101088.s012.docx]

## Table S2 The sequences of primers use in this study.

| Name | Sequence (5’ to 3’) | Tm (℃) |
| --- | --- | --- |
| Vimentin-F | CTC CAC GAA GAG GAA ATC CA | 60 |
| Vimentin-R | GCT TCA ACG GCA AAG TTC TC | 60 |
| N-cadherin-F | GAC AAT GCC CCT CAA GTG TT | 60 |
| N-cadherin-R | CCA TTA AGC CGA GTG ATG GT | 60 |
| GAPDH-F | GAG TCA ACG GAT TTG GTC GT | 60 |
| GAPDH-R | TTG ATT TTG GAG GGA TCT CG | 60 |
| E-cadherin-F | TGC CCA GAA AAT GAA AAA GG | 60 |
| E-cadherin-R | GTG TAT GTG GCA ATG CGT TC | 60 |
| Snail-F | ACC CCA CAT CCT TCT CAC TG | 60 |
| Snail-R | TAC AAA AAC CCA CGC AGA CA | 60 |
| Slug-F | CTT TTT CTT GCC CTC ACT GC | 60 |
| Slug-R | GCT TCG GAG TGA AGA AAT GC | 60 |
| ZEB2-F | CAA CTC CGA TGA ACT GCT GA | 60 |
| ZEB2-R | AGC CTG AGA GGA GGA TCA CA | 60 |
| ZEB1-F | TGC ACT GAG TGT GGA AAA GC | 60 |
| ZEB1-R | TGG TGA TGC TGA AAG AGA CG | 60 |
| Twist1-F^*^ | TCT GGA GGA CCT GGT AGA GG | 55 |
| Twist1-R^*^ | GGA GTC CGC AGT CTT ACG AG | 55 |
