# Supplementary material for: The Tetraindole SK228 Reverses the Epithelial-to-Mesenchymal Transition of Breast Cancer Cells by Up-Regulating Members of the miR-200 Family
Source: PLoS One. 2014 Jun 26;9(6):e101088. doi: 10.1371/journal.pone.0101088 (PMC4072721; doi:10.1371/journal.pone.0101088)
Supplement: Table S3 — Sequences of real-time primers and probes used in this study. (DOCX) [file pone.0101088.s013.docx]

## Table S3 The sequences of real-time primers and probes use in this study.

| Name | Sequence (5’ to 3’) |
| --- | --- |
| miR-200a-F | CGG CGT AAC ACT GTC TGG TAA |
| miR-200a- UPL RT primer | GTT GGC TCT GGT GCA GGG TCC GAG GTA TTC GCA CCA GAG CCA ACA CAT CG |
| miR-200b-F | CGG CGT AAT ACT GCC TGG TAA |
| miR-200b- UPL RT primer | GTT GGC TCT GGT GCA GGG TCC GAG GTA TTC GCA CCA GAG CCA ACT CAT CA |
| miR-200c-F | TCG CTT AAT ACT GCC GGG TAA T |
| miR-200c- UPL RT primer | GTT GGC TCT GGT GCA GGG TCC GAG GTA TTC GCA CCA GAG CCA ACT CCA TC |
| miR-200 Universal reverse primer | GTG CAG GGT CCG AGG T |
| miR-200-TaqMan Probe | Universal ProbeLibrary, Probe #21 |
| RNU6B-F | TCG CTT CGG CAG CAC AT |
| RNU6B-R | AAA AAT ATG GAA CGC TTC ACG AAT |
| RNU6B-TaqMan probe | FAM-TGG AAC GAT ACA GAG AAG ATT AGC ATG GCC-BBQ |
